# Supplementary material for: Machine Learning-Assisted FTIR Analysis of Circulating Extracellular Vesicles for Cancer Liquid Biopsy
Source: J Pers Med. 2022 Jun 10;12(6):949. doi: 10.3390/jpm12060949 (PMC9224706; doi:10.3390/jpm12060949)
Supplement: Supplementary file 1 [file jpm-12-00949-s001.zip › jpm-1746454-supplementary.pdf]

Supplementary Materials

Machine Learning-Assisted FTIR Analysis of Circulating Extracellular Vesicles for Cancer Liquid Biopsy

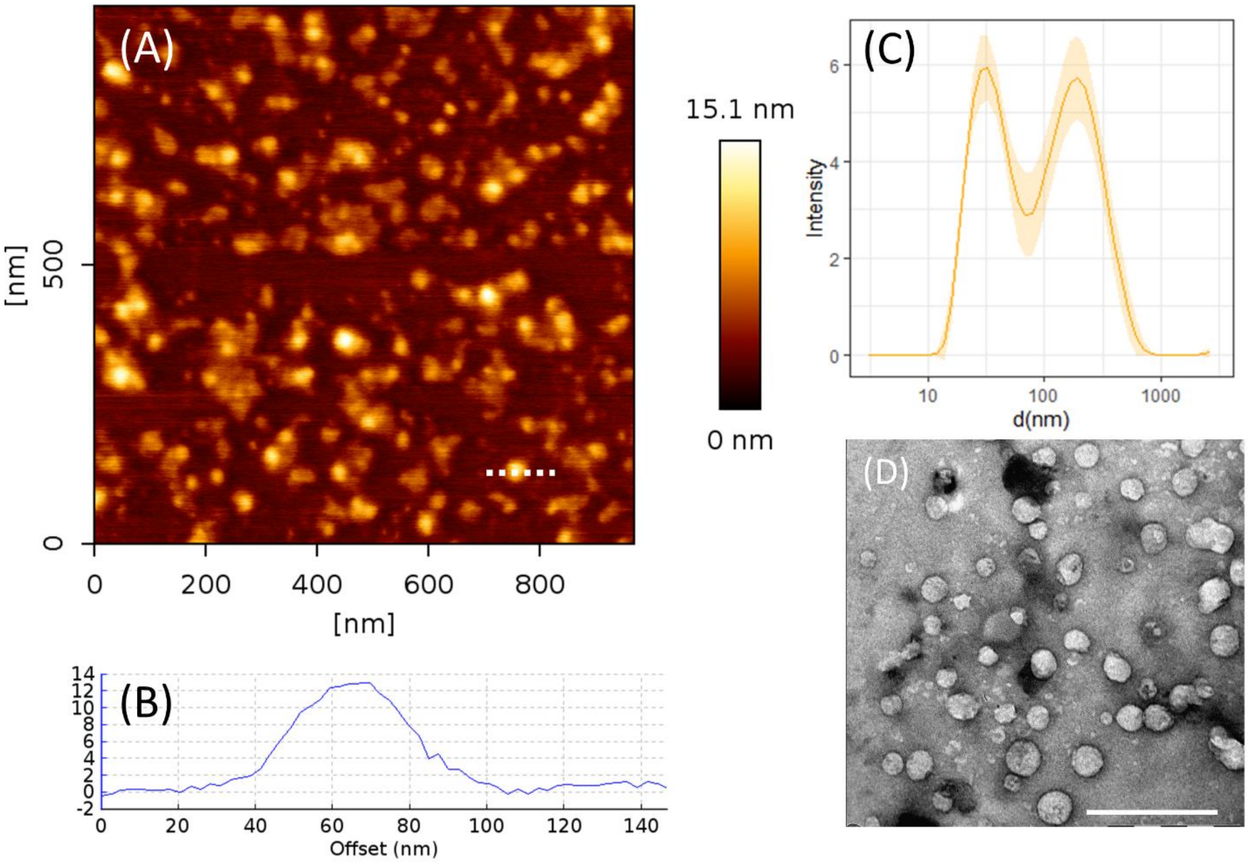

Figure S1: (A) microscopic characterization of a representative purified sample performed with atomic force microscopy. The image is acquired on an EV-enriched suspension extracted from a pool of sera of 5 out of 19 controls recruited in the study. Several similar images were acquired, showing reproducible features. The image shows a large number of rounded particles with the expected size distribution for an EV sample measured with AFM on mica. Particles also display the typical cup shape morphology as shown by the selected **(B)** line profile. Additionally, smaller and partly aggregated particles can be observed, which could be due to the presence of contaminants, such as lipoproteins or albumin. **(C)** An Intensity-weighted diameter distribution measured with DLS. Measurements are acquired on 5 out of the 19 EV-enriched suspensions obtained from control subjects. Measures are reported as mean together with the 95% confidence bands. Two peaks can be observed, one cantered at approximately 32 nm and the other at approximately 190 nm. The first peak is compatible with the average size observed in the AFM image. Moreover, one can observe that microscopic images do not show particles with sizes comparable to the larger peak. This was expected as DLS Intensity data are weighted with the six power of the particle radius, which implies that approximately one over 60.000 particle is as great as 200 nm. Similar size comparison can be detected in the **(D)** TEM image (scale bar = 200 nm). TEM measurements were not acquired on the same samples recruited in the study, but they were purified using the same protocol, thus providing a further confirmation that despite contaminants, the kit allows a substantial enrichment in EVs.
